# Brand public opinion data analysis method based on deep learning

**DOI:** 10.1371/journal.pone.0339563

**Published:** 2025-12-23

**Authors:** Min Li, Wonjun Chung

**Affiliations:** 1 Nanjing Audit University Jinshen College, Nanjing, China,; 2 Department of Design, Tongmyong University, Busan, Republic of Korea; Zhejiang Gongshang University, CHINA

## Abstract

With the rapid development of Internet information technology and digital technology, various network platforms and media are showing a vigorous growth momentum. The powerful power of online public opinion has an immeasurable impact on brand awareness, consumption attitude, and decision-making of consumer groups. For brand owners, responding to sudden online public opinion has become an important issue and a new challenge in the current era. This article mainly focuses on Weibo comments, selects specific brand A, and tracks topic data related to public opinion events of brand A within a certain time range. The emotion dictionary is crucial in analyzing public opinion events using the Latent Dirichlet Allocation (LDA) topic model. This study aims to enhance the emotion dictionary by employing algorithms that leverage topic words and benchmark words, specifically in the context of Dalian University of Technology. The effectiveness of the improved emotion dictionary will be demonstrated through its integration with pre-trained word vectors, utilizing a Bidirectional Encoder Representations from Transformers (BERT) linear sentiment classification model. By combining these methods, the study seeks to provide more accurate sentiment analysis and deeper insights into public opinion. Finally, sentiment orientation analysis is conducted.

## 1. Introduction

With the development of the information age, China’s vast internet user base has formed the world’s largest and most dynamic digital network society. This has given rise to many social platforms and commodity trading platforms with a massive user base and a focus on online dissemination. These platforms have become the primary site for public expression of views, serving as a primary driver of the emergence of online discourse and contributing to the frequent occurrence of diverse opinions in the digital space [1–4]. At present, there is no unified consensus in the academic community on the definition of public opinion. In essence, public opinion can be defined as the collective expression of a phenomenon or problem in real society by netizens through the medium of the internet. This expression takes the form of emotions, wishes, attitudes, opinions, beliefs, dissemination, and interaction, and is driven by the dissemination of information and the interaction of individuals. These characteristics lead to several defining features of online public opinion. Firstly, it exhibits strong suddenness and is difficult to predict. Secondly, after being triggered, public opinion spreads and interacts through multiple pathways. Thirdly, in the virtual world, the identities of Internet users are often hidden, making them challenging to identify and track. Additionally, network information is updated in real time and can be disseminated widely, allowing public opinion to spread infinitely. Finally, the content of online public opinion is rich and diverse [5,6]. Public opinion events that rely on online platforms often spread rapidly across the entire network within a short period. When netizens are inundated with information, they frequently rush to express their opinions and attitudes without careful consideration. Many users tend to convey exaggerated views humorously and emotionally to attract more attention [7,8]. For example, online roast, expression packs, special characters, etc., are widely used, and emotional appeals and flashy surface entertainment occupy a dominant position in the formation of online public opinion. The flashy surface entertainment refers to entertainment activities or content that seem flashy and eye-catching, but are often of low intrinsic quality and value, lacking depth and substance. Based on the above characteristics, the main characteristics of current online public opinion dissemination can be summarized as impatient viewpoints and entertaining emotional demands.

Handling public opinion has become an important task that brands constantly face. In the context of the “Internet+” era, the concept of Internet integration represents a significant development opportunity for traditional industries. By leveraging the potential of Internet technology and innovative thinking, traditional industries can undergo a transformation and upgrade, leading to enhanced efficiency, cost reduction, and the creation of new business models and formats. Therefore, the channels for the public to supervise the brand are more diversified, and the network exposure significantly increases the potential risks of brand b [9]. Brands need to utilize new media and respond to emerging online public opinion in a targeted manner to mitigate negative impacts. The ability to fully utilize online platforms and leverage their role in brand communication and public opinion response has become a significant challenge for brands, as it directly impacts their reputation and future development. In short, online public opinion plays an important role in brand strategy. There is a fundamental difference between the current and traditional public opinion environment. In the context of the contemporary internet, the ability of corporate brands to respond effectively to online public opinion represents a significant challenge [10–12]. The potential impact of public opinion on brand reputation, operations, and even the stability of entire industries cannot be underestimated [13,14].

This study focuses on analyzing the main public opinion risks hidden in brand public opinion data by collecting brand-related data. The objective is to provide comprehensive and multi-dimensional guidance for brand public opinion management and crisis response through the application of deep learning methods.

## 2. Related work

### 2.1. Theme analysis

The topic analysis is a statistical model that does not require language restrictions or manual annotation of data and can extract hidden semantic information from a large amount of text. Theme mining has always been a hot research topic for industry scholars. Dumais et al. [15] proposed a new indexing and retrieval method called Latent Semantic Indexing (LSI). Subsequently, Chappelier et al. [16] introduced the implicit semantic indexing model into probability and proposed the Probabilistic Implicit Semantic Index (pLSI). However, Blei et al. [17] overcame the shortcomings of pLSI and proposed the famous Latent Dirichlet Allocation (LDA). At present, LDA models play an important role in topic modeling and are suitable for short-text topic modeling. This conforms to the short-text characteristics of Chinese Weibo comments and has been widely applied in topic analysis in the Weibo field.

Griffiths et al. [18] proposed a method for determining document content, which states that each document is generated by selecting a topic distribution and then selecting each word from the topics selected according to that distribution. Then, a Markov chain Monte Carlo algorithm was introduced for inference in this model. The algorithm was used to analyze the articles of PNAS, and the number of topics was determined through Bayesian model selection. Newman et al. [19] proposed a novel thematic coherence assessment task that evaluates the coherence or interpretability of a set of words generated by a thematic model. The author applied a series of topic rating models, utilizing WordNet, Wikipedia, and Google search engines, as well as existing vocabulary similarity/correlation studies, to conduct evaluation tasks. Wang et al. [20] introduced a topic model called Topic n-grams, which differs from a traditional bag of words model in that it can discover topics and topic phrases. This model generated word order in text and could capture the meaning of phrases. Chang et al. [21] introduced a novel quantitative approach for measuring semantic meanings in inferred topics, substantiated by extensive user studies. Their findings illustrated that this method was capable of capturing specific elements of the model that previous model quality assessment techniques based on likelihood preservation were unable to discern. Surprisingly, topic models that perform better in preserving likelihood may infer fewer topic meanings.

### 2.2. Sentiment analysis

Sentiment analysis is a technology that draws upon a multitude of research fields, including machine learning, natural language processing, and others. The principal objective of this approach is to discern the emotional proclivities that are obscured within textual data sets of considerable magnitude. This enables the extraction of pertinent information, which is then utilized to ascertain whether the sentiments expressed by users are positive, negative, or neutral.

The research on sentiment analysis can be traced back to the 1980s. However, at that stage, the knowledge structure and technical level of sentiment analysis were not yet perfect, and the research results of scholars were relatively few and scattered [22]. However, with the rapid development of the economy, the growing power of the Internet, the change in social structure, and the improvement of people’s living standards, the channels for obtaining and providing feedback information are increasing. Moreover, the speed of information dissemination is also greatly accelerated. At this stage, sentiment analysis gradually becomes a research hotspot for scholars in various fields [23]. At present, there are a large number of online users on online platforms, which generate a large amount of real-time text data with rich semantics and context, making it very suitable for sentiment analysis. Therefore, extracting different emotional categories from user text data has become a significant research hotspot for scholars. Current research methods primarily include sentiment lexicon-based analysis and sentiment analysis techniques based on machine learning and deep learning.

At this stage, scholars mainly focus on analyzing and researching content such as comments from netizens, social news, and sudden public opinion events. Schuller et al. [24] explored the task of sentiment analysis, which utilizes a combination of machine learning, information retrieval, and natural language processing techniques to identify the polarity and subjectivity of documents. The study placed the problem within the scope of statistical machine learning and investigated and compared different feature selection methods, dimensionality reduction algorithms, and classification techniques. The main focus of the work was to identify the factors that affect the accuracy of the learning model. Extensive statistical analysis was conducted to determine the optimal algorithm configuration. Cambria et al. [25] developed a novel intelligent engine for open domain viewpoint mining and sentiment analysis by integrating graph mining and multidimensional dimensionality reduction techniques onto two common knowledge bases. Socher et al. [26] introduced how to systematically address the semantic word space being unable to express the meaning of longer phrases. Especially in tasks such as emotion detection, understanding combinations required richer supervised training and evaluation resources, as well as more powerful combination models. “Understanding combinations” is the ability to accurately capture the overall meaning of multiple words or phrases when they are combined. In the task of emotion detection, the meaning of a single word may not be able to fully express the emotional tendency of the whole sentence, so it is necessary to consider the combinatorial relationship between words. Tang et al. [27] introduced how to address the challenge of document level sentiment classification, which involves encoding the intrinsic relationships between sentences in the semantic meaning of a document. To this end, a neural network model was introduced to learn vector-based document representations in a unified bottom-up manner. The model first used convolutional neural networks or long short-term memory networks to learn sentence representations. A gated recurrent neural network was used to adaptively encode the semantics of sentences and their relationships in document representation. Document-level sentiment classification experiments would be conducted using four large-scale comment datasets from the Internet Movie Database (IMDB) and the Yelp dataset challenges. Zhu et al. [28] introduced a Three Channel Graph Attention Network (TC-GAT) to capture semantic, syntactic, and multi-faceted dependency information. In addition, a simple and effective fusion mechanism was proposed to comprehensively integrate these three types of information, and the results verified the effectiveness of the proposed model. Shi et al. [29] proposed a new Aspect-Based Sentiment Analysis (ABSA) model, namely Instruction Optimized Graph Convolutional Network (ITGCN). This combination facilitated the advancement of sentiment analysis techniques in practical applications, particularly in the analysis of user feedback pertaining to specific product features or services. The objective was to achieve sub-tasks that predict emotional polarity, thereby revealing underlying contextual information and supporting the prediction of emotional polarity. Mostafa et al. [30] proposed a consumer brand sentiment analysis method based on text mining technology to explore consumers’ feelings toward well-known brands. They used a combination of qualitative and quantitative methods to analyze brand reviews. The results showed that consumers generally held a positive attitude toward several well-known brands. To understand the impact of brand reputation on customer trust and loyalty, and to explore how brands can maintain their reputation, Kayakuş et al. [31] proposed an evaluation method based on machine learning and sentiment analysis. This method collected statistical data of customer reviews, used feature extraction algorithms to extract features from user reviews, and used support vector regression based on sentiment analysis to classify customer product evaluations. The results showed that this method could effectively reveal brand reputation.

A review of the existing literature on brand public opinion analysis reveals a predominant focus on single methods of sentiment analysis or topic mining. There is a notable absence of systematic research that integrates these two approaches in a unified framework. In addition, traditional sentiment analysis methods often rely on pre-built sentiment dictionaries. This limits the applicability of the model in complex contexts and cannot effectively capture consumers’ different emotional responses to details such as brand and product ingredients. This paper studies the adaptability in the face of network buzzwords and a changing public opinion environment. By introducing the combination of LDA topic model and Bidirectional Encoder Representations from Transformers (BERT) linear emotion classification model, the accuracy and robustness of emotion analysis are improved, and the dynamic evolution process of brand public opinion is deeply explored. This research method offers a more comprehensive perspective, which can effectively address the challenges of public opinion management faced by modern brands. Furthermore, it contributes to the development of theory and practice in brand management and marketing. The purpose of this study is to solve the blank problem that the brand public opinion analysis model is single and lacks emotion analysis. The innovation of this study lies in expanding the sentiment lexicon by combining Term Frequency-Inverse Document Frequency (TF-IDF), TextRank, and Second Order Pointwise Mutual Information (SO-PMI), incorporating trending online terms and professional terminology. It addresses the problem that general sentiment lexicons struggle to cover emerging online words and domain-specific terminology. An analysis framework combining LDA topic evolution and BERT emotional classification is constructed to track the dynamic changes of brand emotional topics. At the same time, high-precision real-time emotion judgment is performed, overcoming the limitations of existing methods in emotion-theme joint analysis in the time context. In addition, combining topic keywords with baseline emotion words optimizes the weight of words in the emotion dictionary, making emotion computing more context-aware. Compared with a single model or traditional vocabulary method, it has higher accuracy and robustness in the complex and changing network language environment.

## 3. Improvement of data processing and emotion dictionary

This section mainly introduces the collection and processing of raw brand data, as well as the construction of an emotional dictionary. The comments of a certain brand under the hot search topic on Weibo serve as the source of brand data. By capturing relevant comments on the Weibo topic “Brand A”, relevant topic data within a certain time range are collected. The reason for choosing this brand is that the products of this brand have aroused extensive attention and feedback among consumers, which is suitable for public opinion analysis. Secondly, brand A generates a wealth of public opinion events and discussions on social media, providing sufficient data sources for sentiment analysis. After the data collection is completed, the next step is to perform basic tasks such as data cleaning, deleting stop words, and Chinese word segmentation. After these processes are completed, the processed data are subjected to re-statistical analysis. Then, emotional vocabulary is selected, and keyword extraction algorithms are used to identify representative keywords that accurately reflect the current comment content from the captured topics, ensuring that these keywords convey emotional meaning. The final step is to expand the emotional vocabulary. By collecting and summarizing internet buzzwords, adding keywords, and related algorithms, the selected Dalian University of Technology emotion dictionary is fully expanded.

### 3.1. Brand data collection and preprocessing

**Fig 1:**
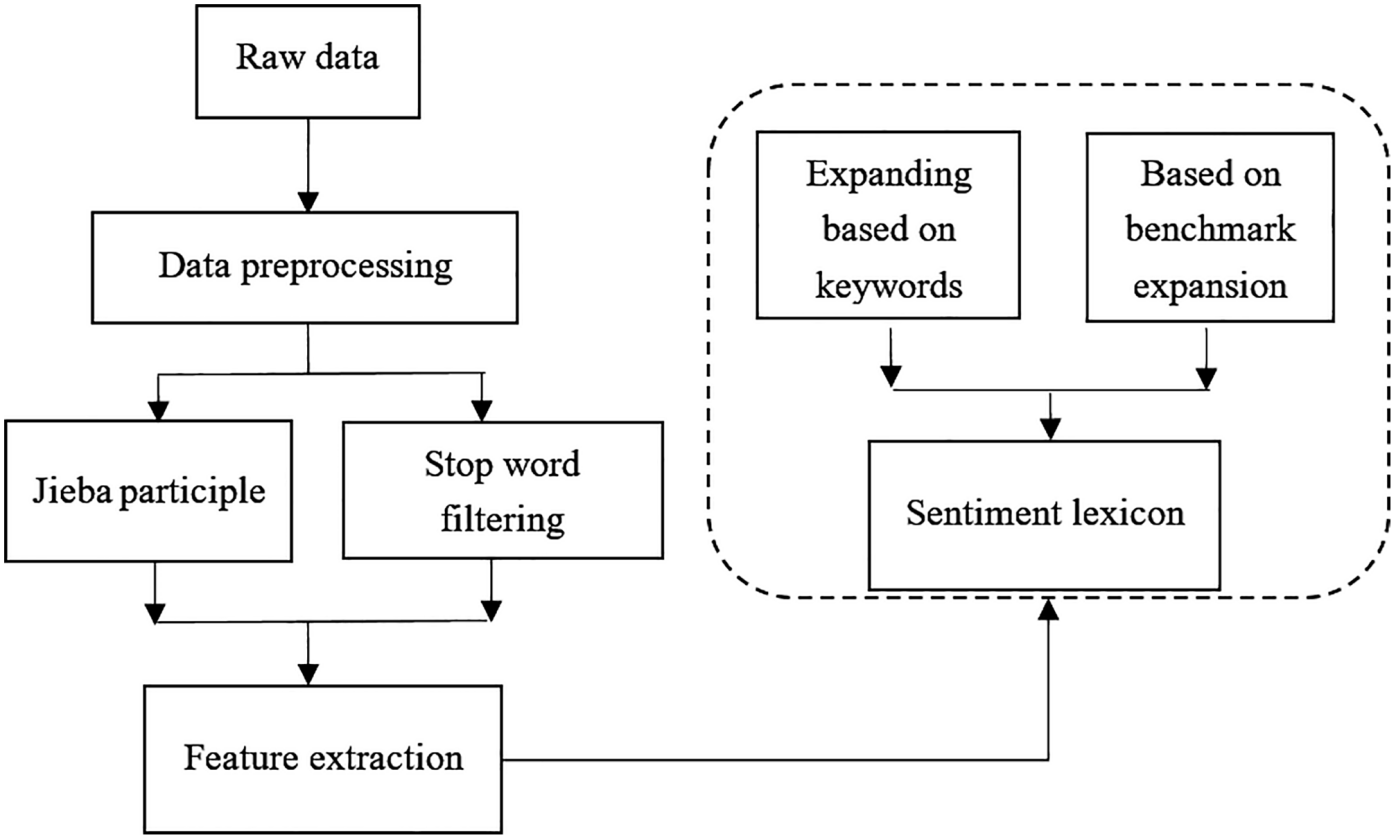
Data processing flow chart.

The core steps of this chapter can be divided into three parts. Firstly, the Python crawler tools are used to collect topic comment data within a certain time range. Secondly, the collected data are segmented, and basic steps such as removing stop words are taken. Finally, statistical analysis and summary of the processed valid data are conducted. The data processing flow is shown in Fig 1.

#### 3.1.1. Data sources.

The research objective of this article is to select a specific brand A, track the topic data related to public opinion events of brand A within a certain time range, and analyze the public opinion events. To achieve this goal, there are three requirements when selecting topic data: (1) Ensure sufficient discussion volume of the data. To conduct subsequent research work, it is necessary to ensure that the selected topic data have sufficient discussion volume. The analysis of discussion hotspots and changes in the topics that netizens pay attention to can only be conducted effectively when the amount of data reaches a certain level. This allows for the enrichment of research content from multiple dimensions and perspectives. (2) Ensure that public opinion topics have a continuous discussion time span. When selecting data, it is important to choose public opinion topics with longer fluctuations to crawl relevant comments at different stages and better grasp the temporal periodicity of public opinion trends. (3) Ensure that public opinion topics are closely related to daily life and have practical significance. The selected public opinion topics should be closely related to people’s daily lives and have certain practical significance, which can provide a reference for brand stakeholders.

After considering several key indicators such as data quantity, data quality, and data update frequency, it is decided to obtain data from the Weibo social media platform. Weibo is a real-time platform where users can quickly post new content and comments. Although the amount of data on Weibo may be very large, there are also kinds of noisy data and information unrelated to the brand. Therefore, in the subsequent data screening and analysis process, efforts will be made to extract useful public opinion information. The data collection and processing process will comply with Weibo’s usage rules, respect user privacy, and platform regulations. Web crawling technology is used to extract brand related data from web pages and strictly comply with relevant data privacy laws and regulations. Meanwhile, measures have been taken to protect users’ privacy information. First, text cleaning of Weibo comments is carried out through automated scripts to identify and delete content that may contain personal information, such as user names, personally identifiable information, and contact information. Secondly, natural language processing is used to detect and block keywords and phrases related to users’ private information. In addition, before releasing data, it is necessary to ensure that all comments are anonymous to avoid association with specific users or their identities. The compliance of data collection and analysis is ensured to avoid infringement and violations.

#### 3.1.2. Data collection.

**Fig 2:**
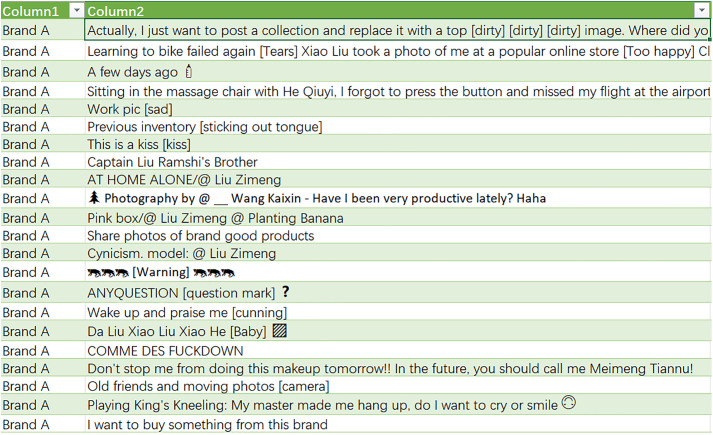
Partial content display.

With the rapid development of the Internet and the acceleration of network information transmission, the traditional search engine pages can no longer meet people’s daily needs, so many more convenient web interfaces have emerged. This article uses the Weibo topic search interface and Python programming to collect comment data on topics related to “Brand A”. By repeatedly crawling the risk review comments and public opinion status of Brand A during different time periods, the comment data shown in Fig 2 are obtained.

#### 3.1.3. Data preprocessing.

There is some invalid and meaningless content in the original data, such as “forwarding Weibo”, “clicking on links to receive red envelope coupons”, and some irrelevant comments with relevant topic labels. These data are irrelevant to the research presented in this article and may skew the results of subsequent emotional analyses. Therefore, it is essential to preprocess the raw data prior to conducting the experiment.

First, during the data cleansing process, automated text processing techniques are investigated to efficiently remove “garbled, repetitive, meaningless, and irrelevant” content. Specifically, for garbled data, character encoding detection tools are used to identify and eliminate characters or phrases that cannot be parsed. The elimination of duplicate content is achieved by calculating the text hash to ensure that only unique comments are retained. To filter out meaningless and irrelevant content, a custom keyword filtering list is used, combined with natural language processing technology to delete comments that are not related to the research topic. These rules and methods are generic and can be extended to subjects other than “Brand A” to ensure standardization and consistency in data cleaning, thereby improving the accuracy of all types of sentiment analysis. Secondly, it is necessary to filter out stop words and retain the effective content after data cleaning. Due to the diversity of Weibo comments and the wide range of topic discussions, the existing set of stop words cannot fully meet the needs of all data. Weibo comments contain many invalid characters, such as “@”, “|”, “#”, etc. Therefore, it is necessary to remove these invalid characters from the comments. To avoid mistakenly deleting brand-related comments, a combination of manual sampling and semantic similarity verification is used. 1,000 filtered comments are randomly selected and manually judged by 3 annotators to determine whether they are related to the brand. For filtering meaningless content, for example, in an original comment “Forwarding Weibo//@User: Brand A’s new product is amazing!”, “Forwarding Weibo” is a meaningless action and is removed after being identified by the keyword filtering list, leaving “Brand A’s new product is amazing!” as the valid comment. For filtering irrelevant content, for example, in an original comment “#Brand A# The weather is so nice today, perfect for hiking”, although it contains the #Brand A# tag, the core content is unrelated to the brand and is therefore judged as irrelevant and removed. For filtering garbled content, for example, in an original comment “Brand A ¥$ products are really good ¥$”, “¥$” is an unparseable garbled character and is deleted after detection, leaving “Brand A products are really good”. This article selects Baidu’s stop word list and expands it based on actual Weibo comment data, customizing the content of stop words. A detailed list of stop words can be found in Table 1, which can be roughly divided into two categories: English and symbols.

**Table 1:** List of discontinued words in Weibo comments (Partial).

Category	Discontinuation words (partial)
English	In, and, the, am, also, at, by, is, few, theirs, below, weren’t
symbol	“!”,”?”,”@”,”“,”~”,”*”,”¥”,”$”,”{“,”]”,”③”,”φ”,”㈧”,”&”

Finally, text segmentation is performed on the cleaned and filtered valid data. Due to the complex logic and contextual expression of Chinese compared to other languages, simply changing the position of participles can make the meaning of a sentence completely different. The term “segmentation processing,” as employed in this article, refers to the act of dividing a valid comment into discrete words, each of which possesses a distinct meaning. These words are then assembled in the original word order, thereby preserving their inherent semantic nuances. The Jieba tokenization library in Python is used for preprocessing text data. In practical operation, it is found that many proprietary terms and internet buzzwords are used in the comments, but these words are not stored in the Jieba segmentation library, so incorrect segmentation results may occur in experimental operations. In user reviews, sometimes brand names are connected to other words, and word segmentation algorithms may separate them, leading to misunderstandings. For example, “Coca-Cola” is divided into “Coca” and “Cola”, rather than being used as a whole vocabulary. To avoid this situation, Jieba’s user-defined dictionary feature is used to add “Coca Cola” as a custom vocabulary to ensure they are correctly segmented. The user-defined dictionary created is shown in Table 2, which is mainly divided into two categories: Internet hot topics and professional words.

**Table 2:** Jiaba word segmentation custom dictionary.

Category	Words (Part)
Internet hot topics	Divine horses are all floating clouds, true fragrance, autumn waves, rising postures, kneeling, happy to be a father, green tea bitch
Specialized word	Meituan, Ele.me, Coca Cola, Meinianda, Didi, SF Express

Finally, using Weibo as the data platform, Brand A’s relevant data for three month are collected. During the data collection phase, 200,000 comments are obtained. After removing 100,000 irrelevant comments and undergoing Jieba segmentation and manual screening, 37,234 valid data are obtained.

### 3.2. Expansion and improvement of emotional dictionary

In the initial sentiment analysis task, the emotion dictionary plays a crucial role. This dictionary contains a large number of emotional words and their corresponding emotional polarities, which can help the system accurately identify emotional tendencies in the text. The method, based on sentiment lexicon, supports sentiment classification and sentiment polarity judgment. The system quickly determines whether the emotions expressed in the text are positive, negative, or neutral by matching text vocabulary with emotional words in the emotion dictionary, achieving automated analysis of text emotions. However, emotional dictionaries have limited vocabulary, language is constantly evolving, and new vocabulary and expressions are constantly emerging. Existing emotional dictionaries are difficult to cover all emotional vocabulary and expressions. Therefore, expanding the emotion dictionary helps the system better understand and analyze textual sentiment information. Different social and cultural backgrounds, industry fields, and age groups may have specific emotional vocabulary and expressions, and existing emotional dictionaries are difficult to cover all contexts. Expanding the emotion dictionary can make the system more adaptable to different text contexts, improving the applicability and accuracy of sentiment analysis. To improve the accuracy of subsequent sentiment analysis tasks, this article adopts two methods to mine sentiment feature vocabulary and expand the emotion dictionary.

#### 3.2.1. Expanding the emotion dictionary based on subject words.

A keyword extraction algorithm is a technique used to automatically extract keywords from text, which can help people quickly understand the theme and content of the text. Some common keyword extraction algorithms nowadays include TF-IDF, TextRank, etc., which can help people quickly understand the content and topic of text, thereby improving the efficiency of text processing and information retrieval. This article plans to use TF-IDF and TextRank to extract keywords from comment data and expand the emotion dictionary.

The TF-IDF algorithm is a widely used technique in text mining and information retrieval for identifying and evaluating keywords or topics in text. This algorithm comprehensively considers the Term Frequency (TF) of a word in the text and its frequency of occurrence in the Inverse Document Frequency (IDF) to determine the importance of the word.

Firstly, the TF of each word in a specific document is calculated, which is the ratio of the number of times the word appears in the document to the total number of words in the document. This can help understand which words are more prominent and important in a specific document. The calculation formula:


$$\begin{array}{c} tf-idf_{i,j}=tf_{i,j}\times idf_{i} \end{array}$$ (1)

In the above equation, $tf-idf_{i,j}$ is the product of tf and idf. The larger the value of the product, the higher the importance of the feature vocabulary. Among them, $tf_{i,j}$ is the ratio of the number of times feature vocabulary appear in comments to the total number of words, as shown in the formula (2):


$$\begin{array}{c} tf_{i,j}=\frac{n_{i,j}}{\sum_{k}n_{k,j}} \end{array}$$ (2)

Secondly, the IDF is calculated, which measures the general importance of a word in the entire document set. The denominator is the number of comments containing feature vocabulary, and the numerator D is the number of all comments. To avoid the denominator calculation result being 0, which means that this word does not exist in the dataset, the value is smoothed by adding 1. The specific formula:


$$\begin{array}{c} idf_{i,j}=\text{log}\frac{D}{\text{1}+\left|j:t_{i}\in d_{j}\right|} \end{array}$$ (3)

If a word frequently appears in many documents, its IDF value will be lower, indicating that the word has a lower ability to distinguish specific documents. On the contrary, if a word appears in a few documents, its IDF value will be higher, indicating that the word has higher importance in distinguishing documents. By multiplying TF and IDF, the TF-IDF value for each word can be obtained. By ranking and analyzing these values, which words are most representative and discriminative in the document can be determined thereby identifying the topic or keywords of the document.

The TF-IDF model is used to calculate the importance of feature vocabulary in the brand data collected for each cycle. The TF-IDF value of each word in the topic is used as the weight value, and the words in the topic are sorted according to the weight value. The top 20 words with the highest weight are selected as keywords. The top 5 TF-IDF weight values are shown in Table 3.

**Table 3:** Ranking of TF-IDF words in brand data.

Rank	Word	Weight value
1	taste	0.342
2	flavor	0.335
3	cheer	0.257
4	support	0.238
5	additive	0.168

Table 3 shows that in the obtained data, consumers are most concerned about the “taste” and “flavor” of brand A, and there is no significant difference in the importance of these two words. Secondly, the ranking of the third and fourth words shows that consumers generally hold a supportive attitude towards the brand. The fifth word in the final ranking is “food additive”, which indicates that consumers are very concerned about this indicator of the product. It also reminds the brand that the most relevant indicator to consumers is additive, which facilitates subsequent guidance on product optimization and quality improvement. Analysis shows that the application of the TF-IDF algorithm can quickly and accurately identify key themes and brand-related keywords in brand data. It can add the keywords corresponding to the first 20 weight values identified to the emotion dictionary, providing strong support for brand public opinion analysis and information retrieval.

#### 3.2.2. Expansion of emotion dictionary based on benchmark words.

As a cultural phenomenon in contemporary society, network catchwords reflect people’s communication ways and thinking patterns on the Internet and social media. With the popularity of the Internet, online catchwords are widely spread in social platforms and online communities dominated by microblogs. However, due to its unique language characteristics and cultural connotations, it may confuse unfamiliar people.

Since the raw data come from Weibo, to improve the accuracy of sentiment analysis, the experimental plan is to use the SO-PMI method to construct a network popular language dictionary and integrate it into the emotion dictionary. SO-PM is a method used to evaluate the correlation between words, typically applied in natural language processing and information retrieval. It is an extension of the traditional Pointwise Mutual Information (PMI) concept, as shown in (4).


$$\begin{array}{c} PMI\left(x,\;y\right)\;=\;\text{log}\frac{P\left(x,\;y\right)}{P\left(x\right)P\left(y\right)} \end{array}$$ (4)

P(x, y) is the probability of both word x and word y appearing simultaneously. P(x) and P(y) are the probabilities of word x and word y appearing independently, respectively. When P(x, y) is larger, it indicates a higher degree of correlation between the two words, and vice versa.

Different from other emotional words, network catchwords spread rapidly on the Internet and can be accepted and used by a large number of people in a short time. However, at the same time, internet slang has a short lifecycle, frequent updates, and strong timeliness. Therefore, this article chooses the network hot words recommended by the Sogou Pinyin input method as candidate word libraries for constructing a popular language dictionary. At the same time, by filtering the crawled Weibo corpus, these new online words can be obtained. This method can not only avoid the tedious work of mining emotional new words from massive Weibo text data but also obtain the latest network hot words. Specifically, this article first selects 42,937 new words from the latest version of the Sogou Pinyin input method as candidate words, and then filters them in the newly crawled Weibo corpus. Sogou Pinyin input method is a widely used Chinese input method tool, with its powerful lexicon and intelligent prediction function, to provide users with a fast and accurate Chinese input experience. Its role in the research is mainly reflected in providing a rich candidate lexicon for the construction of the network buzzword dictionary. Selecting the latest version of hot words helps to improve the accuracy of sentiment analysis and adaptability to contemporary language habits, providing brand public opinion analysis with emotional characteristics that are more closely related to users’ actual expressions. Finally, based on word frequency sorting and manual review, 56 new words with the highest usage frequency that have not yet appeared in the existing emotion dictionary are determined. Next, the emotional polarity of these new words will be determined through benchmark words. Benchmark words usually refer to a set of basic vocabulary used to represent emotional polarity (such as positive or negative emotions), which can help analyze and identify emotional colors in text for sentiment analysis and recognition. The benchmark words are mostly common emotional words, such as “like”, “happy”, “angry”, “sad”, etc. They form the core part of the emotional dictionary and are used to measure the emotional tendency and intensity in the text. By analyzing the frequency of occurrence of vocabulary in the HowNet emotion dictionary in the corpus, a total of 30 pairs of positive and negative reference words are determined, as shown in Table 4.

**Table 4:** Benchmark word list.

Positive benchmark words				
Friendly	Warmth	Honesty	Patience	Rich
Flexibility	Innovation	Happiness	Respect	Goodwill
Comfortable	Elegance	Outgoing	Effort	Reliable
Happiness	Enrich	Bravery	Bright	Hope
Trust	Purely	Harmony	Harmony	Knowledge
Achievements	Excellence	Sweet	Value	Enthusiasm
**Pejorative benchmark term**				
Poor	Pain	Decay	Melancholy	Despair
Grey	Disappointment	Failure	Crushing	Betrayal
Loneliness	Bad habits	Discomfort	Low mood	Embarrassing
Fatigue	Irritable	Disgust	Boring	Bored
Hopeless	Poverty	Unfortunately	Obstacles	Despair
Indifference	Frustration	Dishonesty	Procrastination	Injury

Finally, the sentiment polarity of internet slang is calculated using formula (4). The resulting dictionary contains a total of 56 internet slang words, including 30 positive sentiment words and 26 negative sentiment words. The selected emotional words are extended to the emotional dictionary, and some vocabulary is shown in Table 5.

**Table 5:** Partial popular internet slang.

Part of speech	Internet slang
Positive emotional words	Super awesome, amazing, steady, necessary, awesome…
Negative emotional words	Tearing, roast, throwing the pan, overturning…

#### 3.2.3. Improvement of the emotion dictionary.

In the emotional dictionary of Dalian University of Technology, each word has two important attributes: emotional strength and emotional polarity. Therefore, it is necessary to add the expanded keywords and popular phrases to the original dictionary and merge them into the Weibo domain emotional dictionary. Some emotional words are shown in Table 6.

**Table 6:** Part of the new words in the emotional dictionary.

New words	Emotional intensity	Polarity
Super thumbs up	5	1
Awesome	3	1
Catfight	7	2
Throwing the pot	3	2

Among them, emotional intensity includes five levels, namely 1, 3, 5, 7, and 9; The polarity of emotional words involves three categories: neutral, positive, and negative, with corresponding values of 0, 1, and 2. This article makes further improvements based on the Dalian University of Technology Emotion Dictionary, setting the weight of feature vocabulary in the non-Dalian University of Technology Emotion Vocabulary Ontology Library to 1, and adding 1 to the strength corresponding to other emotion feature vocabulary on the original basis. Thus, it ensures both the addition of emotional feature vocabulary to the classification model and the influence of non-emotional feature vocabulary on the classification model. The “influence of non-emotional feature vocabulary on classification models” is considered. While these words lack explicit emotional polarity, they can influence the overall emotional tendency of a text in a specific context. The emotional intensity of the new word is estimated by combining the emotional polarity of the existing emotion dictionary with the relative emotional intensity based on the reference word. Specifically, for each new network buzzword, its context and actual use in public opinion data are first analyzed, and the emotional polarity of the relevant benchmark words is compared one by one. The benchmark words include positive and negative emotion words, such as “like” and “angry”. The new words are associated with the corresponding benchmark words according to the emotional performance of these benchmark words in the public opinion discussion. The formula for calculating the emotional strength of the final feature vocabulary:


$$\begin{array}{c} s\left(w\right)=v\left(w\right)p\left(w\right)\ldots \end{array}$$ (5)

v(w) represents the emotional intensity of the vocabulary. p(w) is the emotional polarity of the vocabulary. s(w) is the emotional value of the vocabulary. In formula (5), to calculate the strength and polarity of a set of words, “strength” is first defined as a numerical value representing the emotional strength conveyed by the word in sentiment analysis, usually obtained from scores in sentiment dictionaries. Emotional polarity:


$$\begin{array}{c} p\left(w\right)==\frac{\sum_{i=\text{1}}^{N}\rho \left(A,x\right)}{N} \end{array}$$ (6)

p(w) represents the emotional polarity of the new word, N represents the number of words, A represents the new word, x represents the benchmark word, and ρ represents the correlation degree with the new word. If the emotional tendency of the new word is the same as that of the base word, its emotional polarity is more positive or negative. The emotional intensity of new words:


$$\begin{array}{c} v\left(w\right)=\sum_{i=\text{1}}^{N}\rho (A,x)∙V(x) \end{array}$$ (7)

V(x) represents the emotional value of the reference word. The study assumes that the emotion value of “like” is 5 and “anger” is −5. If “super like” is used 10 times with “like” and 2 times with “anger” in a comment, and the total number of occurrences of “super like” in all texts is 1000 times, then the emotional intensity of the new word “super like” is calculated as (0.01*5)+(−5*0.002)=0.04.

These scores can be calibrated according to how positive or negative the emotion is. For example, positive emotion words such as “excellent” may have high intensity values, while negative emotion words such as “bad” have negative values. The study combines the strength and polarity of all the words and obtains the combined emotional strength and polarity of the whole group of words by weighted summing each word. This calculation method can effectively reflect the emotional tendency of the whole sentence or phrase, and then be used in the task of emotion classification.

## 4. Brand public opinion theme analysis

Both Weibo data and the vocabulary found in sentiment dictionaries are rich in connotations and exhibit strong flexibility in conveying meaning and emotions. The same sentiment can be expressed in various forms, which can pose challenges for traditional methods. Therefore, this chapter analyzes brand public opinion themes from two aspects: public opinion analysis based on the LDA theme model and emotion classification based on the BERT model.

### 4.1. Brand public opinion analysis based on LDA theme model

**Fig 3:**
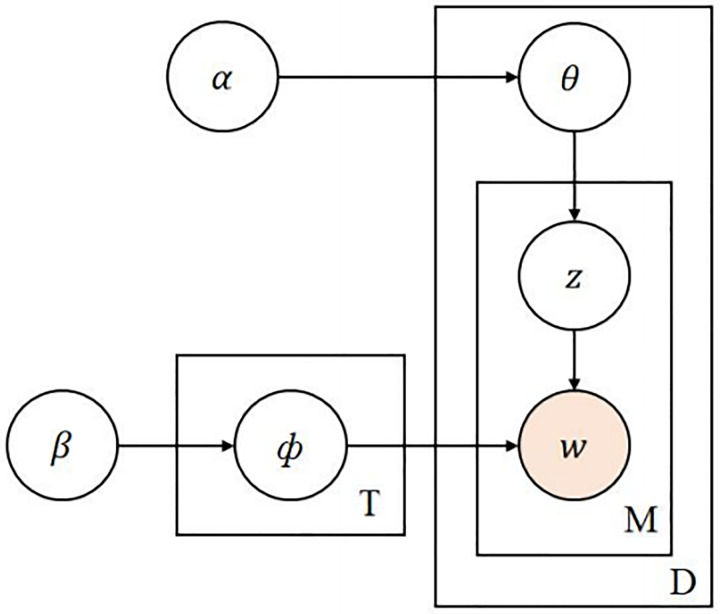
LDA theme model.

This section mainly explores the evolution of the theme brand’s public opinion. LDA topic modeling is a commonly used text mining technique used to discovering potential topic structures in document collections. To check whether the performance difference is significant, the study uses t-test for data analysis. Articles can be represented through topic distribution, and each topic distribution can be represented by the distribution of vocabulary. In public opinion analysis, LDA can help us understand the evolution and changes of public opinion topics. The specific implementation is shown in Fig 3.

θ is the polynomial distribution of the theme and z is the theme corresponding to the words in the brand review data. *ɸ* is the polynomial distribution of words and w is the randomly selected feature vocabulary from the polynomial of words. Through LDA, each document in the document collection can be represented as a mixture of a set of topics, each topic consisting of a vocabulary. Through this method, potential themes in the document can be discovered, and the relationships and evolutionary trends between different themes can be understood. In public opinion analysis, LDA can be used to explore the evolution and changes of public opinion themes in different periods or events.

The aim of this study is to explore the evolution patterns of keywords and themes of the “Brand A” topic in different stages of public opinion development. For this purpose, an unsupervised learning LDA topic model is used for modeling and analysis. The LDA topic model has significant advantages in this study, as it can effectively process text and extract a more representative feature vocabulary, thereby mining more refined topics. According to the lifecycle theory of online public opinion, brand-related online public opinion is divided into three stages. The first stage involves calculating the confusion level in the LDA topic model and determining the number of topics. An expanded vocabulary is integrated with a custom Jieba dictionary to ensure that expanded terms are not split during word segmentation. A bag-of-words containing all expanded terms is then constructed. By testing the confusion level corresponding to different numbers of topics, the number of topics with the lowest confusion level is selected as the optimal configuration. The second stage, based on the determined number of topics, repeats the process of expanding the bag-of-words to construct the LDA input for the fermentation stage text data. After modeling, topic keywords are extracted, and topic differentiation characteristics are analyzed. The third stage performs the same expanded vocabulary integration operation on the stable stage text data, completes the LDA model, and compares the changes in topic distribution across the three stages. Then, based on the results of the LDA topic model, public opinion topics for each stage are extracted and analyzed to reveal the evolution of topics.

Firstly, in the data preprocessing stage, stop words in the data are segmented and removed to establish a stored segmentation list. The segmentation of each piece of data is iterated. The comments are marked as a word list, and punctuation marks and other characters are removed to organize the text data, as shown in Table 7.

**Table 7:** Word segmentation list.

Organized text
[‘Delicious’], [‘Dishes’], [‘Always’], [‘Let people’], [‘Intoxicated’], [‘No matter’], [‘Yes’], [‘Chinese food’], [‘Or’], [‘Western food’], [‘Both’], [‘Have’], [‘Each’], [‘Features’], [‘For example’], [‘neneneba Kung Pao Chicken’], [‘Spicy’], [‘Delicious’], [‘Let people’], [‘Salivating’], [‘And’], [‘Pizza’], [‘Then’], [‘Soft’], [‘Pasta’], [‘Plus’], [‘Rich’], [‘Ingredients’], [‘Letting people’], [‘Can’t stop’], [‘In addition’], [‘Also’], [‘Dessert’], [‘Cake’], [‘Ice cream’], [‘All’], [‘Perfect’], [‘Select’], [‘Party’], [‘Time’], [‘Share’], [‘Food’], [‘More’], [‘Enhance’], [‘Emotion’], [‘Important’], [‘Way’], [‘Every mouthful’], [‘All’], [‘Right’], [‘Life’], [‘Enjoy’], [‘Let’s’], [‘Together’], [‘Explore’], [‘More’], [‘Delicious’], [‘Bar’], [‘...’]

To reduce the number of words in the corpus, only words that appear more than 5 times are used. Then, the data are organized into a format suitable for the LDA model. Finally, the LDA topic model is established, the perplexity is calculated, and the number of topics is determined. Confusion is the degree of uncertainty that the topic model obtained after training has about the topic to which a document belongs, and this difficult to determine difference is called perplexity. The lower the perplexity, the lower the uncertainty, and the more advantageous the topic model is for topic clustering. The formula for calculating perplexity is as follows:


$$\begin{array}{c} perplexity\left(test\right)=\;\text{exp}\left\{-\frac{\sum_{d=\text{1}}^{M}logp\left(w_{d}\right)}{\sum_{d=\text{1}}^{M}N_{d}}\right\} \end{array}$$ (8)

$$\begin{array}{c} p\left(w\right)=\;p\left(z\;|d\right)*\;p\left(w\;|z\right) \end{array}$$ (9)

Among them, M represents the number of texts in the corpus. $N_{d}$ represents the number of words in the d-th document. $P(W_{d})$ represents the probability of the text. p(z|d) represents the distribution of text d on topic z. For the word *w* in the bag of words model, p(w) is the product of the distribution values of the word in all topics and the distribution of the topic where the word is located.

### 4.2. Brand data sentiment classification model based on BERT model

**Fig 4:**
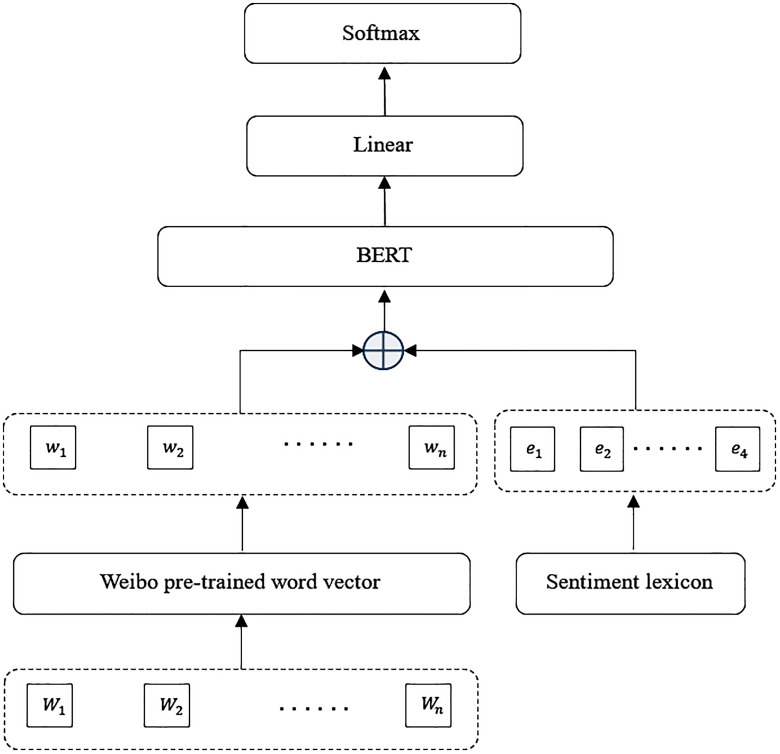
The sentiment classification model based on BERT.

Analyzing textual emotions can intuitively reflect the distribution of netizens’ preferences and emotions towards brands. There are currently multiple methods for sentiment classification. This study focuses on the emotions triggered by “Brand A” in online public opinion, and the emotions in comment texts mainly include positive emotions, negative emotions, and neutral emotions (objective emotions). Traditional methods typically involve cleaning and segmenting the obtained public opinion text, then comparing it with an emotion dictionary, scanning one by one to identify and extract sentiment words from the text. By accumulating the scores corresponding to each sentiment word, the sentiment value of the paragraph is finally calculated, and the sentiment classification result is obtained. Based on the extraction of topic features by LDA, this study introduces BERT to construct a topic-enhanced BERT sentiment classification model, integrating the topic evolution process into the sentiment judgment process. Through feature fusion, the model can perceive the thematic context to which the current text belongs while understanding the semantics, thereby enhancing its ability to recognize emotional expressions. The sentiment classification model based on BERT is shown in Fig 4.

As shown in Fig 4, BERT is a model based on the Transformer [32] encoder. A linear classifier is added at the top of the BERT [33] model to identify positive, negative, and neutral emotions contained in each brand’s public opinion data. The Chinese microblog data Weibo pre-trained word vectors provided by the NLPIR Lab laboratory is used to vectorize the processed segmented text $W_{i}=\left\{W_{\text{1}},W_{\text{2}},\ldots ,W_{n}\right\}$ to obtain the vector representation $w_{i}=\left\{w_{\text{1}},w_{\text{2}},\ldots ,w_{n}\right\}$. Then, the sentiment feature vector with w_i is fused and sent to the BERT for feature extraction. Then the last representation ${\text{h}}_{i}=\left\{{\text{h}}_{\text{1}},{\text{h}}_{\text{2}},\ldots ,{\text{h}}_{n}\right\}$ is input into the linear layer, and a softmax operation is performed to obtain the probability distribution on the relation, as shown in (10–12).


$$\begin{array}{c} {\text{h}}^{*}\;=\;Linear\left(Q{\text{h}}_{i}+b\right) \end{array}$$ (10)

$$\begin{array}{c} \hat{\text{p}}\left(\text{y}|\text{S}\right)=\text{softmax}\left({\text{W}}^{\left(\text{S}\right)}{\text{h}}^{*}+{\text{b}}^{\text{S}}\right) \end{array}$$ (11)

$$\begin{array}{c} \hat{\text{y}}\;=\text{arg}\underset{\text{y}}{\text{max}}\hat{\text{p}}\left(\text{y}|\text{S}\right) \end{array}$$ (12)

*Linear* represents the linear transformation layer. Q represents the weights of the linear transformation layer. b represents the bias of the linear transformation layer. $\hat{\text{p}}\left(\text{y}|\text{S}\right)$ represents the probability distribution of the input S belonging to each sentiment category. ${\text{h}}^{*}$ represents the feature vector after passing through the linear layer. ${\text{W}}^{\left(\text{S}\right)}$ and ${\text{b}}^{\text{S}}$ represent the weights and biases of the Softmax classification layer. Through the processing of the Softmax function, the value of each node output by the model will be mapped to a probability, representing the confidence level of the corresponding emotional category for that node. When predicting, the category with the highest probability is used as the final sentiment classification result, and y ^is the final sentiment label obtained. The BERT-based model mainly embeds information by combining sentiment words and tweet vectors through linking or weighted aggregation. Firstly, BERT embeddings are generated for each tweet comment, which capture deep semantic information about the text. Meanwhile, improved emotional vocabulary is used to generate embeddings of emotional features. Considering that the words in the sentiment lexicon require a general semantic representation independent of specific contexts, the pre-trained static word vector models Word2Vec and Global Vectors for Word Representation (GloVe) are used to obtain the basic vector representations of sentiment words. Unlike the context-dependent embeddings generated by BERT, GloVe generates static word vectors by decomposing the global word-word co-occurrence matrix, which can capture the global semantic information of words. GloVe and Word2Vec, as alternative static word vector schemes, are used to generate a fixed vector representation for each sentiment word in the sentiment lexicon. They provide a basic semantic embedding for each word in the sentiment lexicon, which can complement the dynamic context representations generated by BERT. The specific expression:


$$\begin{array}{c} {\text{E}}_{\text{emotional},\text{j}}=\text{Word2Vec}/\text{GloVe}({\text{A}}_{\text{j}}) \end{array}$$ (13)

$$\begin{array}{c} {\text{E}}_{\text{i}}=\text{Bert}({\text{C}}_{\text{i}}) \end{array}$$ (14)

${\text{E}}_{\text{emotional},\text{j}}$ represents the emotional word vector, ${\text{C}}_{\text{i}}$ represents the number of brand products. These emotional features provide information about the emotional tendencies in the comments. When integrating these two types of information, a connection method is used to concatenate the embeddings generated by BERT with the embeddings of emotional features in the feature dimension, forming a richer representation. Specifically, the document-topic distribution vector of the comment is obtained using a trained LDA model. Then, the semantic embedding vector of the comment is obtained using a BERT model, and the sentiment feature vector is calculated using an improved sentiment lexicon. Next, feature fusion is performed, concatenating the topic distribution vector, the BERT semantic vector, and the sentiment feature vector to form the final fused feature representation. Finally, this fused feature vector is fed into a linear classification layer and an activation function layer for final sentiment classification. To ensure the reproducibility, all code, preprocessed data, and key experimental results have been publicly released on GitHub. The experiments are conducted on a hardware environment consisting of an Intel Core i9-12900K CPU @ 3.20GHz, an NVIDIA RTX 4090 GPU (24GB VRAM), and 32GB of DDR5 memory, running Ubuntu 20.04 LTS, and using Python 3.8.10, PyTorch 1.13.1, Transformers 4.28.0, Gensim 4.2.0, and Scikit-learn 1.1.3. The pseudo-code is shown in Table 8.

**Table 8:** Pseudo-code for public opinion data analysis.

Pseudo-code
# Dependencies: torch, transformers, jieba, sklearnimport requests, jieba, numpy as npfrom sklearn.decomposition import LatentDirichletAllocationfrom transformers import BertForSequenceClassification, Trainer, TrainingArguments # 1. Crawl Weibo commentsdef crawl(): return requests.get(“Weibo API URL”, headers={”User-Agent”: “...”}, timeout = 10).json()[“data”] # 2. Preprocess: clean + segment textdef preprocess(txt): return “ “.join([w for w in jieba.cut(re.sub(r”[^\u4e00-\u9fa5]”, ““, txt)) if w not in stopwords]) # 3. Expand emotion dictionary (TF-IDF + SO-PMI)def expand_dict(corpus): return {**origin_dict, **{w: {“intensity”:3,”polarity”:1} for w in tfidf_top_words(corpus)}} # 4. LDA topic modeling lda = LatentDirichletAllocation(n_components = 3, random_state = 42).fit(tfidf_matrix)# 5. BERT sentiment classification (fuse topic features) model = BertForSequenceClassification.from_pretrained(“bert-base-chinese”, num_labels = 3) trainer = Trainer(model, args = TrainingArguments(...), train_dataset = dataset) trainer.train()print(“Full workflow completed: Crawling→Preprocessing→Dict Expansion→LDA→BERT Classification”)

## 5. Experimental result

### 5.1. LDA theme model public opinion analysis results

“Topic non-overlap” means that in multiple topic models established on the same data set, the content and features represented by the identified topics are clearly distinguished without crossover or overlap. This means that each topic focuses on a specific aspect or topic and does not share similar keywords or concepts with other topics. When analyzing Weibo comments, the topic can be a discussion formed around different aspects of Brand A (such as product quality, customer service, price satisfaction, etc.). Each topic contains specific keywords that reflect the user’s opinions and emotional tendencies. Then, based on the results, keywords for each stage theme are extracted. To display the results, 5 keywords are selected for each topic.

Table 9 shows that taste has always been ranked high in high-frequency keywords, indicating that the taste of Brand A’s products is always the most important selling point and the most concerned point for consumers. As time goes by, consumers are increasingly concerned about the additive ingredients of Brand A products. This analysis suggests that Brand A may have been detected to contain a certain degree of preservatives and other additive components. The ranking of keywords such as “encourage”, “cheer”, and “support” has begun to fluctuate, indicating that some consumers may be able to accept Brand A containing compliant doses of anti-corrosion additives. Others cannot accept Brand A containing additive ingredients. Therefore, with the discussion of online public opinion, the weight of Brand A’s “nutrition” keywords has gradually decreased with the dissemination among consumers.

**Table 9:** Different topic theme high frequency words in three stages of brand public opinion communication.

	Topic 1	Topic 2	Topic 3
1	taste	taste	taste
2	nutrition	flavor	flavor
3	flavor	cheer	additive
4	encourage	preservative	support
5	preservative	nutrition	nutrition

By analyzing the results of the keywords corresponding to the topics generated by the LDA model, it is found that the changes between different topics reveal the complex psychology of consumers between brand trust and product quality. They not only hope to enjoy delicious products but also strive to ensure the safety and health of the food they consume. Overall, Brand A needs to pay more attention to the transparency of product ingredients while maintaining its taste advantage, to meet the needs of different consumers.

To further analyze the relationships between words, this study constructs a co-occurrence network matrix based on high-frequency keywords from three stages and calculates the co-occurrence frequency of core keywords. Co-occurrence refers to the simultaneous appearance of keywords in the same review. A higher co-occurrence frequency indicates a stronger correlation between the consumer’s attention dimension represented by the keyword. The results are shown in Table 10.

**Table 10:** Keyword co-occurrence frequency matrix.

Keywords	Taste	Flavor	Nutrition	Additives	Preservatives	Support	Cheer	Encouragement
Taste	/	1286	892	345	412	568	492	387
Flavor	1286	/	976	489	523	612	589	421
Nutrition	892	976	/	278	305	456	398	324
Additives	345	489	278	/	867	213	189	156
Preservatives	412	523	305	867	/	245	201	178
Support-	568	612	456	213	245	/	732	689
Cheer	492	589	398	189	201	732	/	598
Encouragement	387	421	324	156	178	689	598	/

Table 10 shows that “taste” and “tasting” has the highest co-occurrence frequency, reaching 1,286 times, reflecting consumers’ high attention to taste experience in product evaluation. “Taste” and “Tasting” together constitute the taste experience word pair, becoming the most concentrated dimension of consumer discussion. Additives and Preservatives have the second highest co-occurrence frequency, reaching 867 times. This is because both fall under the category of food ingredient safety, and consumers often strongly associate these two concepts, forming a safety concern word pair. Pairs of words of support and cheers and pairs of words of support and encouragement also contribute significantly, reaching 732 and 689 times, respectively, indicating a strong correlation with positive emotions. These results indicate that product experience and safety concerns are the two core association clusters, corresponding to the themes identified by LDA. Positive emotion words are linked around these two, meaning that different themes are associated through the cross-co-occurrence of core keywords, thus covering the dimensions of consumer discussion about the brand.

### 5.2. Experimental results of data sentiment classification

#### 5.2.1. Evaluation indicators.

In natural language processing, evaluation metrics are used to assess the performance of a model or system in processing natural language tasks. To more accurately measure the performance of the model, appropriate evaluation criteria need to be selected for different tasks and models [34]. The sentiment classification experiment of this article uses Precision, Recall, and F-value as evaluation metrics, with the specific formula as (15)-(17):


$$\begin{array}{c} P\;=\;\frac{\text{TP}}{\text{TP}+\text{FP}} \end{array}$$ (15)

$$\begin{array}{c} \text{R}=\;\frac{\text{T P}}{\text{TP}+\text{FN}} \end{array}$$ (16)

$$\begin{array}{c} F\;=\;\frac{{\text{2P}}^{*}\text{R}}{\text{P}+\text{R}} \end{array}$$ (17)

In the above formula, TP (true cases) is the number of samples correctly predicted as positive categories. FP (false positive cases) is the number of samples incorrectly predicted as positive categories. FN (false negative cases) is the number of samples incorrectly predicted as negative categories. TN (true negative cases) is the number of samples correctly predicted as negative categories.

Accuracy is the simplest evaluation metric, which represents the proportion of samples correctly predicted by the model to the total sample. It is commonly used to compare the learning performance of different deep learning models. Accuracy refers to how many samples predicted as positive are true examples. The recall rate represents the proportion of successful predictions made by the model in the actual case. To comprehensively consider precision and recall, the F-value is usually used as the evaluation criterion for model performance, which is the harmonic average of precision and recall. The higher the F-value, the better the model performance.

#### 5.2.2. Experimental results and analysis.

To ensure the authenticity and validity of the experiment, the evaluation benchmark uses manually labeled data. From 37,234 valid data points, 3000 are randomly selected. Three annotators with experience in sentiment analysis independently label the data according to three sentiment categories: positive, negative, and neutral. The final labeling results are determined by majority voting and used as the model evaluation standard. The comparison benchmarks include three classic deep learning models: CNN, LSTM, and BiLSTM, as well as a BERT + linear model without an extended sentiment lexicon, to verify the performance of the proposed method. Precision, recall, and F1 score are used as evaluation metrics. All processed Weibo comment texts are randomly divided and subjected to multiple cross validations to eliminate the impact of a single abnormal experiment on the accuracy of the results. Finally, the average of multiple classification results is taken as the experimental result. Statistical analysis is performed using an independent samples t-test. The significance level is set at 0.05, and *p* < 0.05 is considered statistically significant. The word vector is trained using the Weibo provided by the NLPIR Lab laboratory, combined with the training word vector and emotion feature matrix, with a dimension of 300. The BERT model used is base (L = 12, H = 768, A = 12, Total Parameters = 110 M). The optimization algorithm used is the Adam algorithm for adaptive moment estimation, and a Dropout layer is introduced to reduce the overfitting problem of the neural network. The parameter is set to 0.3. Although the Adam optimizer can adaptively adjust the learning rate, it still needs to continuously adjust the parameters in the experiment to achieve optimal performance. The experimental results are shown in Table 11.

**Table 11:** Performance evaluation of emotion classification algorithm.

Sentiment lexicon	Model	Average precision rate	Average recall rate	Average F1 score	*p*-value compared to a model with the same structure but without an emotion dictionar-y
Not Introduced Emotional Dictionary	CNN	0.691 ± 0.023	0.689 ± 0.025	0.689 ± 0.024	/
	LSTM	0.733 ± 0.019	0.721 ± 0.021	0.726 ± 0.020	/
	BiLSTM	0.865 ± 0.015	0.863 ± 0.016	0.864 ± 0.015	/
	BERT+Linear	0.893 ± 0.012	0.889 ± 0.013	0.891 ± 0.012	/
Introduce Emotional Dictionary	CNN+Emotion Dictionary	0.707 ± 0.021	0.698 ± 0.022	0.702 ± 0.021	0.038
	LSTM+ Emotion Dictionary	0.762 ± 0.018*	0.758 ± 0.017*	0.760 ± 0.017*	0.029
	BiLSTM+ Emotion Dictionary	0.891 ± 0.014*	0.887 ± 0.015*	0.889 ± 0.014*	0.041
	BERT+Linear+ Emotion Dictionary	0.913 ± 0.011*	0.911 ± 0.012*	0.912 ± 0.011*	0.023

Note: * means p < 0.05 compared with without the introduction of emotion dictionary.

According to Table 9, the proposed method has a statistically significant advantage over other methods in sentiment classification, with an average precision of 0.91 ± 0.01, an average recall of 0.911 ± 0.012, and an average F1 score of 0.912 ± 0.011. Compared with the BERT+linear model without an emotion dictionary, the proposed method shows the largest improvement in average precision (0.02), average recall (0.022), and average F1 score (0.021), demonstrating the best performance. The other models listed in Table 9 show an average improvement in precision (0.0014), average recall (0.0146), and average F1 score (0.024). Furthermore, the differences in the three metrics between the models with sentiment dictionaries and the meta-model are statistically significant (*p* < 0.05). Overall, expanding the sentiment lexicon and increasing the weights of sentiment features improve the classification performance of deep learning models. This effect is validated by an independent samples t-test, which eliminates the influence of random fluctuations and further ensures the reproducibility of the experimental results.

In addition, the emotional proportions of three different stages are analyzed. The emotional proportions of brand data identified by the pre-trained model in different stages are shown in Table 12.

**Table 12:** The proportion of emotions included in brand data during different stages.

Sentiment classification	Phase 1	Phase 2	Phase 3
Positive	75.65%*#	70.66%*#	73.45%*#
Negative	11.62%	14.87%	12.67%
Neutral	12.73%	14.47%	13.88%

Note: * means p < 0.05 compared with Negative; # means p < 0.05 compared with Neutral.

The sentiment analysis of brand A’s online public opinion data shows the fluctuation of brand A’s reputation in the minds of consumers. The main reason why the research divides the time period into three parts is to capture the development dynamics of public opinion and the changes in consumer emotions. By subdividing the data time period, the characteristics of public opinion, emotional tendency, and their evolution trajectory at each stage can be observed in more detail. The time phases are delineated according to the temporal period in which the event occurred. The initial phase, which typically occurs during the first week following the event, is characterized by a rapid and intuitive public response. The second phase, the developmental phase, covers one to three weeks after the event, and comments during this phase usually reflect the in-depth discussion and gradual deepening of the public’s feelings about the event. The final phase is the stabilization phase, which typically refers to the period exceeding three weeks after the event. During this phase, comments may reflect more enduring alterations in perceptions and attitudes. In the initial stage, the positive emotions of Brand A account for the vast majority, indicating that consumers hold a good view and evaluation of Brand A. However, with the passage of time and the influence of food additives, there has been an increase in negative emotions in Brand A’s public opinion, with a decrease of about 5% in the proportion of positive emotions and an increase of about 3.2% in negative emotions. Subsequently, Brand A may have taken some positive measures, such as official debunking or scientific clarification, which had a positive impact on consumer emotions. In the third stage, consumers’ positive emotions towards Brand A recover by approximately 2.79%, while their negative emotions decrease by 0.87%. This indicates that the positive image of Brand A is expected to continue to improve in the short term.

These results further demonstrate the importance of sentiment analysis in public opinion monitoring and brand management. Emotion analysis can provide a deeper understanding of consumers’ emotional attitudes towards brands and capture trends in emotional changes promptly. This provides important reference for brand decision-makers, enabling them to take corresponding measures based on changes in public opinion, such as improving product quality, strengthening publicity, and communication, to maintain and enhance brand reputation. From the experimental results and T-test analysis, the model with the emotion dictionary has better performance on different evaluation indexes. After the introduction of the emotion dictionary, the model can identify the emotion tendency in the text more effectively, especially in complex network language and emotion expression. The expansion of the emotion dictionary not only includes web buzzwords and industry-specific terms, but also better reflects consumers’ true feelings towards brands. Through sentiment analysis, the research can further understand the changes of consumers’ attitudes and emotions towards brands, and provide a basis for brands’ strategic decisions and marketing strategies. In the complex and changeable public opinion environment, corresponding measures can be taken to enhance brand image and consumer trust. The three major themes identified by LDA in this study, namely product experience theme, safety concern theme, and emotional attitude theme, have cross-scenario promotion. This framework is applicable to food and beverage brands in the same industry, as well as daily necessities, maternal and child products, and other consumer brands. It is consistent with the logic of public opinion generation that is intuitive and sensitive to the safety attributes of such products. By combining keyword co-occurrence with emotional proportion features, the dynamic changes in brand A’s image can be observed. In the first stage, the initial brand image focuses on high-quality taste experience providers. In the second stage, due to safety disputes, the brand image diverges. In the third stage, the brand image is reorganized into a comprehensive positive image with stable core experience and controllable safety attributes. This confirms that brand image is the result of the dynamic interaction between consumer attention dimensions and emotional tendencies.

In brand sentiment analysis, a comprehensive method combining the LDA topic model and the BERT linear sentiment classification model is proposed. This framework has enabled a more profound examination of the subject matter and emotional proclivities associated with online public opinion, particularly with regard to the analysis of intricate consumer sentiments. This approach has also addressed a notable deficiency in existing research, namely the absence of a combination of multidimensional methods. The study also expands the emotion dictionary, enhances the adaptability to network buzzwords and contemporary contexts, and improves the accuracy of sentiment analysis.

## 6. Conclusion

This study collected and preprocessed Weibo comments related to Brand A through web scraping technology, and optimized the dictionary based on keywords and benchmark words to expand and improve the emotion dictionary. Then, by analyzing the public opinion evolution of Brand A through the LDA topic model, a sentiment classification model integrating the sentiment lexicon BERT linear model was proposed, and its superiority was verified through comparative experiments. The practical significance of the research is that it provides an efficient public opinion management tool for brand owners to help them timely understand the changes in consumer sentiment and its impact on brand image. Through in-depth analysis of public opinion data, brands can develop more targeted coping strategies and marketing plans, thereby optimizing brand image, enhancing consumer trust, and reducing potential public opinion crisis risks. The results of this study have important reference value for brand managers to cope with public opinion challenges in complex and changing market environments. It also provides operational research models and methods for related academic fields.

Under the new network situation and environment, brand public opinion is a topic worthy of continuous research. The limitation of the current research is that it only focuses on a specific Brand A and fails to cover the public sentiment dynamics of other brands or industries, thus limiting the universality of the analysis results. Despite the introduction of Bert-based sentiment analysis models, other deep learning models may perform better in sentiment classification. Therefore, future research should consider expanding the scope and period of brands, combining more diversified models for comprehensive analysis, to have a more comprehensive understanding of the complexity and dynamics of brand public opinion. At the same time, further exploration of the application of multi-modal data in the analysis of brand public opinion will also provide a richer perspective for the research.
